# Estimated Carbon Emissions Savings With Shifts From In-Person Visits to Telemedicine for Patients With Cancer

**DOI:** 10.1001/jamanetworkopen.2022.53788

**Published:** 2023-01-31

**Authors:** Krupal B. Patel, Brian D. Gonzalez, Kea Turner, Amir Alishahi Tabriz, Dana E. Rollison, Edmondo Robinson, Cristina Naso, Xuefeng Wang, Philippe E. Spiess

**Affiliations:** 1 Department of Head and Neck and Endocrine Oncology, Moffitt Cancer Center, Tampa, Florida; 2 Department of Otolaryngology–Head and Neck Surgery, University of South Florida, Tampa; 3 Department of Health Outcomes and Behavior, Moffitt Cancer Center, Tampa, Florida; 4 Department of Cancer Epidemiology, Moffitt Cancer Center, Tampa, Florida; 5 Department of Internal and Hospital Medicine, Moffitt Cancer Center, Tampa, Florida; 6 Center for Digital Health, Moffitt Cancer Center, Tampa, Florida; 7 Virtual Health Program, Moffitt Cancer Center, Tampa, Florida; 8 Department of Biostatistics and Bioinformatics, Moffitt Cancer Center, Tampa, Florida; 9 Department of Genitourinary Oncology, Moffitt Cancer Center, Tampa, Florida

## Abstract

**Importance:**

While the health care community advocates broadly for climate change policy, medical professionals can look within care practices to assess their contribution to carbon dioxide (CO_2_) emissions, and provide solutions wherever possible. Telemedicine can help in mitigating climate change by providing care from a distance.

**Objective:**

To assess the carbon savings achieved from telemedicine visits.

**Design, Setting, and Participants:**

This cross-sectional study of telemedicine visits was conducted at a single-institution National Cancer Institute (NCI)-designated comprehensive cancer center. Eligible patients were aged 18 years and above, completed telemedicine visits from April 1, 2020, to June 20, 2021, and had a Florida mailing address documented in their electronic medical record. Groups were divided between those within driving time of 60 minutes (1-way) to the cancer center vs those living beyond 60 minutes of drive time. Data were analyzed between April 2020 and June 2021.

**Main Outcomes and Measures:**

Carbon emission savings from telemedicine, measured in total and average per-visit savings.

**Results:**

A total 49 329 telemedicine visits with 23 228 patients were conducted from April 1, 2020, to June 30, 2021. A total 21 489 visits were for patients with driving time of 60 minutes or less (median [IQR] age, 62.0 [52.0-71.0] years; 12 334 [57.4%] female; 1685 [7.8%] Black, 1500 [7.0%] Hispanic, 16 010 [74.5%] non-Hispanic White), while 27 840 visits were for patients with driving time greater than 60 minutes (median [IQR] age, 67.0 [57.0-74.0] years; 14 372 [51.6%] female; 1056 [3.8%] Black, 1364 [5.0%] Hispanic, 22 457 [80.7%] non-Hispanic White). For patients living within a driving time of 60 minutes from the cancer center, 424 471 kg CO_2_ emissions were saved (mean [SD] emissions savings, 19.8 [9.4] kg CO_2_ per visit) due to telemedicine—equivalent to 91.5 passenger vehicles driven for 1 year. For patients whose driving distance was greater than 60 minutes, 2 744 248 kg CO_2_ emissions were saved (mean emissions savings, 98.6 [54.8] kg CO_2_ per visit)—equivalent to 591 passenger vehicles driven for 1 year.

**Conclusions and Relevance:**

Using a large data set, this cross-sectional analysis highlighted the carbon emissions savings due to telemedicine in oncology. This has important implications in reducing health care–related carbon footprint.

## Key Points

**Question:**

Can telemedicine visits for patients with cancer help in reducing carbon emissions?

**Findings:**

This cross-sectional study included 49 329 telemedicine visits at a National Cancer Institute (NCI)-designated comprehensive cancer center from April 1, 2020, to June 30, 2021. For patients living within a driving distance of 60 minutes from the cancer center, an estimated 424 471 kg carbon dioxide (CO_2_) emissions were saved (per-visit mean savings of 19.8 kg CO_2_ emissions) due to telemedicine, the equivalent of 91.5 passenger vehicles driven for 1 year. For patients whose driving distance was greater than 60 minutes, 2 744 248 kg CO_2_ emissions were saved (per-visit mean savings of 98.6 kg CO_2_ emissions), the equivalent of 591 passenger vehicles driven for 1 year.

**Meaning:**

These results highlight the carbon emissions savings that could be gained with the increased use of telemedicine in oncology. This has important implications in reducing health care–related carbon footprint.

## Teaser

This cross-sectional study of patients with cancer treated by a comprehensive cancer center in Florida estimates the reductions carbon emissions associated with increased use of telemedicine visits.

## Introduction

In 2020, global carbon dioxide (CO_2_) emissions fell by 6.4%, or 2.3 billion tons, as an unintended effect of the COVID-19 pandemic’s restrictions.^1^ The US led the reduction in emissions with a nearly 13% decrease; this was mostly due to decreases in transportation,^1^ which is currently the largest source of greenhouse gas (GHG) emissions in the US after having surpassed electricity generation in 2016.^2^ From 2008 to 2018, US health care sector GHG emissions rose by 6%, approximately 553 metric tons of CO_2_ equivalent emissions, or 8.5% of domestic GHG emissions.^3^ Per capita CO_2_ equivalent emissions were 1693 kg, the highest among industrialized nations. Recently, more than 200 leading health journals published a joint commentary on the current climate emergency, with a call for urgent action to reduce the impact of climate change on health.^4^ This statement was published in anticipation of the 26th United Nations Climate Change Conference of the Parties. While the health care community advocates for climate change policy, we must also look within care practices and assess our contribution to the CO_2_ emissions and provide solutions wherever possible.^5,6^ While previous studies have focused on smaller patient populations in the prepandemic era, telemedicine was rapidly adopted during the COVID-19 pandemic to improve widespread access to health care.^7^ Not only does telemedicine provide substantial cost benefits and improve access, but it can also help in mitigating climate change by providing care from distance.^8,9^ In this study, we used a large oncology patient data set to estimate the CO_2_ emission savings from implementing telemedicine at Moffitt Cancer Center (MCC), a National Cancer Institute (NCI)-designated cancer center.

## Methods

### Study Sample

This was a retrospective study of patients seen at MCC, an NCI-designated comprehensive cancer center in Florida. Due to the COVID-19 pandemic, implementation of telemedicine at MCC was accelerated in March 2020. Telemedicine was defined as real-time care delivered through a synchronous videoconferencing. Starting in April 2020, MCC instituted a synchronous video platform (Zoom Meetings) for telemedicine visits. All telemedicine visits with a mailing address listed in the electronic medical record within the State of Florida from April 1, 2020, to June 30, 2021, were included in the study. All patients were offered telemedicine if deemed appropriate by the clinical team. Telemedicine visits were not offered to patients who needed physical examinations beyond what can be assessed during a telemedicine visit. This study was deemed exempt from review by the MCC institutional review board with a waiver of informed consent from patients due to low risk. Baseline demographics data including age, sex, insurance, race and ethnicity were abstracted from electronic health record. The Strengthening the Reporting of Observational Studies in Epidemiology (STROBE) reporting guideline for cross-sectional study was used as a reference to report study design and findings.

### Statistical Analysis

All patients with addresses within the State of Florida were assumed to travel round-trip via an automobile from the home address listed in the electronic medical record to MCC as a final destination. For patients with a post office box as their mailing address, their zip code’s centroid was used as driving departure point. All patients included in the study had addresses on record.

Driving distance traveled in miles was calculated in October 2021 by an analytics organization (Buxton Company) that uses Alteryx’s analytic platform to provide geospatial data. Briefly, the locations were geocoded, and the distance between the 2 geocoded locations was calculated by finding the route that resulted in the least amount of drive time.

CO_2_ emissions saved for vehicle travel were calculated using EPA emissions calculator, which estimates 411 g of CO_2_ emissions per vehicle per mile traveled.^10^ Analyses were completed and maps were generated using the tmap^11^ package in R version 4.2.0 (R Project for Statistical Computing). CO_2_ emissions equivalencies were determined using EPA equivalencies calculator.^12^

## Results

From April 1, 2020, to June 30, 2021, 49 329 telemedicine visits (23 228 patients) were for patients residing within the same state as MCC (Florida). The majority of the patients coming to MCC were within 60 minutes of 1-way driving time (eFigure 1 in Supplement 1). Thus, subgroups were divided based on driving time of 60 minutes or less vs greater than 60 minutes for further analysis to determine CO_2_ emissions saved between the 2 groups. A total 21 489 visits (10 027 patients) were for patients living less than or equal to 60 minutes 1-way driving distance from MCC, and 27 840 visits (13 201 patients) were for patients living more than 60 minutes of driving distance from MCC (Table 1). For those with visits within 60 minutes of driving time, median (IQR) age was 62.0 years (52.0-71.0 years), 12 334 (57.4%) of the visits were female patients, and 9934 (46.2%) of the visits were by patients privately insured; 1685 (7.8%) were for Black patients, 1500 (7.0%) for Hispanic patients, and 16 010 (74.5%) for non-Hispanic White patients. For those with visits with greater than 60 minutes of driving time, median (IQR) age was 67.0 years (57.0-74.0 years), 13 468 (48.4%) of the visits were female patients, and 10 217 (36.7%) of the visits were by patients privately insured; 1056 (3.8%) were for Black patients, 1364 (5.0%) for Hispanic patients, and 22 457 (80.7%) for non-Hispanic White patients.

**Table 1.** Demographics of Telemedicine Visits at Moffitt Cancer Center

Characteristics	Visits, No. (%)	
	Driving time ≤60 min (n = 21 489)	Driving time >60 min (n = 27 840)
Total patients	10 027	13 201
Age, median (IQR), y	62.0 (52.0-71.0)	67.0 (57.0-74.0)
Sex		
Female	12 334 (57.4)	14 372 (51.6)
Male	9155 (42.6)	13 468 (48.4)
Insurance		
Private	9934 (46.2)	10 217 (36.7)
Medicare	9434 (43.9)	15 321 (55.0)
Medicaid	1102 (5.1)	1017 (3.7)
Others	1019 (4.7)	1285 (4.6)
Race and ethnicity		
Black	1685 (7.8)	1056 (3.8)
Hispanic White	1500 (7.0)	1364 (5.0)
Non-Hispanic White	16 010 (74.5)	22 457 (80.7)
Other^a^	2294 (10.7)	2963 (10.6)

^a^ Including Asian, American Indian, and Native Hawaiian or other Pacific Islander.

For patients who lived within a driving distance of 60 minutes from MCC, an estimated 1 032 775 round-trip miles were saved as a result of telemedicine, corresponding to an estimated 424 471 kg of CO_2_ in emissions savings (Table 2, Figure; eFigure 2 in Supplement 1). Per-visit mean (SD) savings of 48.1 (22.1) miles and 19.8 (19.4) CO_2_ kg emissions were noted. For patients whose driving distance to MCC was greater than 60 minutes, 6 677 002 roundtrip miles were saved, corresponding to an estimated 2 744 248 kg of CO_2_ in emissions savings (Table 2, Figure; eFigure 2 in Supplement 1). Per-visit mean (SD) savings of 239.8 (84.2) miles and 98.6 (54.8) CO_2_ kg emissions were noted. Overall, patients who lived greater than 60 minutes of driving distance from MCC had approximately 6 times more savings in CO_2_ emissions and subsequent equivalent number of passenger vehicles driven for 1 year compared with those who lived within 60 minutes of driving distance (91.5 kg for ≤60 minutes vs 591 kg for >60 minutes), gallons of gasoline saved (47 763 gal for ≤60 minutes vs 308 794 gal for >60 minutes), home electricity use for 1 year (82.6 homes for ≤60 minutes vs 534 homes for >60 minutes), home energy use for 1 year (53.5 homes for ≤60 minutes vs 346 homes for >60 minutes), number of tree seedlings grown for 10 years (7019 for ≤60 minutes vs 45 376 for >60 minutes), and carbon sequestered by acres of US forests in one year (502 for ≤60 minutes vs 3248 for >60 minutes).

**Table 2.** Estimated CO_2_ Emission Savings From Reduced Driving Emissions Due to Patients With Cancer Using Telemedicine

Characteristic	Driving time^a^	
	≤60 min	>60 min
No. of patients	10 027	13 201
No. of visits	21 489	27 840
Round-trip driving distance saved, mi		
Total	1 032 775	6 677 002
Per visit		
Mean (SD)	48.1 (22.8)	239.8 (133.3)
Median (IQR)	49.0 (30.0-65.0)	204.0 (148.0-302.0)
CO_2_ kg emissions saved		
Total	424 471	2 744 248
Per visit		
Mean (SD)	19.8 (9.4)	98.6 (54.8)
Median (IQR)	20.1 (12.3-26.7)	83.8 (60.8-124.1)
Equivalent GHG emissions for passenger vehicles driven for 1 y, No. of vehicles	91.5	591
Equivalent CO_2_ emissions		
Gasoline consumed, gal	47 763	308 794
Home electricity use for 1 y, No. of homes	82.6	534
Home energy use for 1 y, No. of homes	53.5	346
Equivalent carbon sequestration		
Tree seedlings grown for 10 y	7019	45 376
Acres of US forests in 1 y	502	3248

Abbreviations: CO_2_, carbon dioxide; GHG, greenhouse gas.

^a^ All drive times are based on 1-way trips unless otherwise noted.

**Figure.**
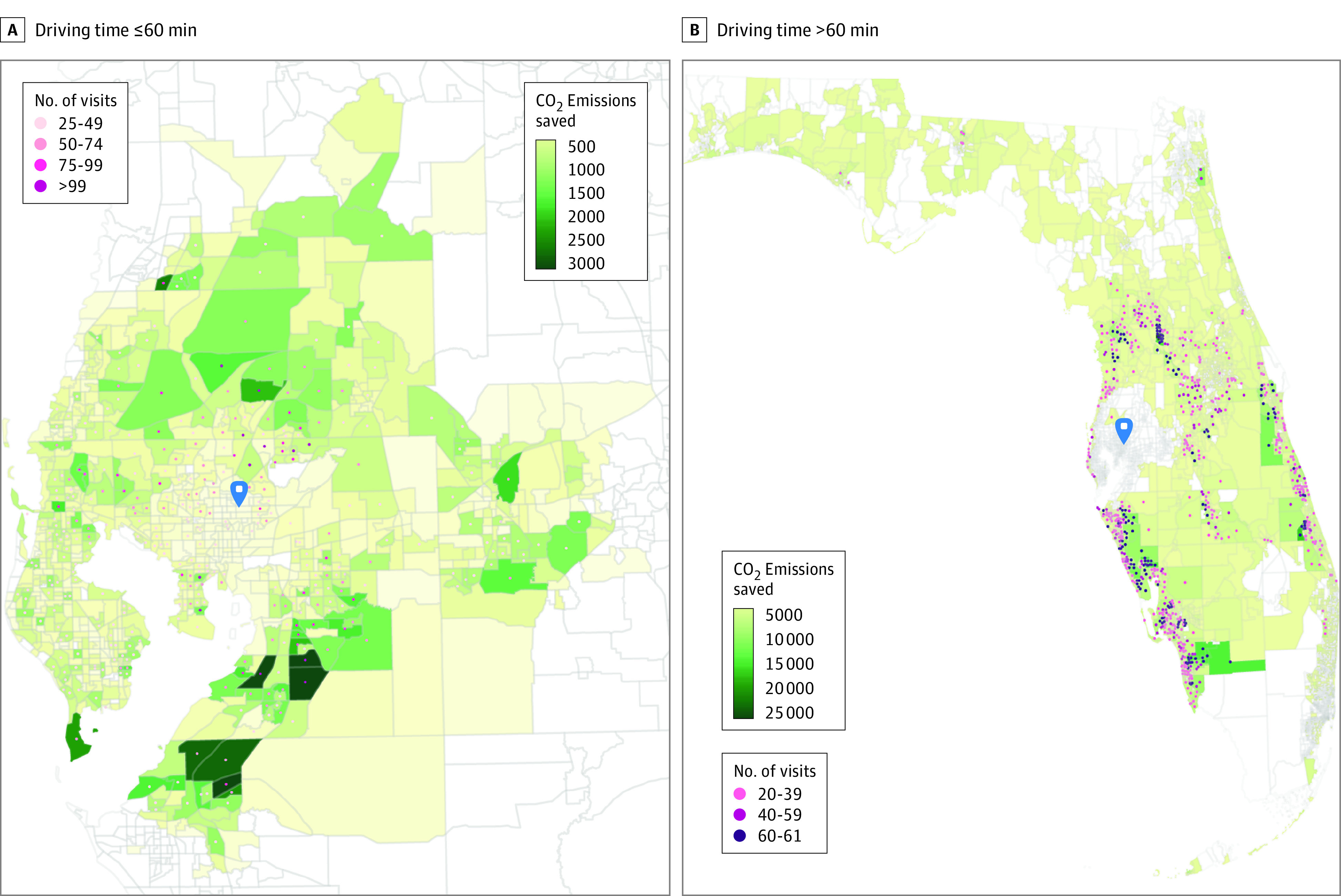
Carbon Dioxide (CO_2_) Emissions Saved From Telemedicine Visits The blue marker indicates the Moffitt Cancer Center. Geographical boundaries represent census tracts; census tract centroids were used to map the number of visits per tract. In total, fewer than 25 visits are not shown in panel A, and fewer than 20 visits are not shown in panel B.

We additionally analyzed carbon emissions savings based on the lower (ie, 386 g/mi) and upper (435 g/mi) limits of emissions per mile (eTable 1 in Supplement 1).^13^ For the lower limit of emissions per mile, patients who lived within a driving distance of 60 minutes from MCC saved an estimated 398 651 kg of CO_2_ emissions, with mean (SD) savings of 18.6 (8.8) kg of CO_2_; based on the upper limit of emissions per mile, an estimated 449 257 kg of CO_2_ emissions were saved with mean savings of 20.9 (9.9) kg of CO_2_. Using the lower limit of emissions per mile, patients whose driving distance to MCC was greater than 60 minutes saved an estimated 2 577 323 kg of CO_2_ emissions with mean savings of 92.6 (51.5) kg of CO_2_; while using the upper limit of emissions per mile, an estimated 2 904 496 kg of CO_2_ emissions were saved with mean savings of 104.3 (58.0) kg of CO_2_.

## Discussion

In this cross-sectional study using a large data set, implementation of telemedicine was estimated to result in substantial savings in carbon emissions due to driving. While previous studies have been limited to smaller sample sizes,^14,15,16,17,18,19,20,21,22,23^ our study included a large sample size and focused on oncology patients when large-scale telemedicine implementation was undertaken during COVID-19 pandemic. Telemedicine can help address diagnostic and treatment delays and improve access to high-quality care,^18,19,20,21,22,23^ as seen during COVID-19 pandemic. Telemedicine also provides significant advantages for patients who live farther away from treatment centers by improving access to care, reducing financial toxic effects, and subsequently reducing CO_2_ emissions.^14,15,16,17^ There has been a reduction in the number of rural hospitals over the previous decade, consequently almost doubling the number of people who live outside a 60-minute driving range of a major hospital.^24^ As a result of rural hospital closures, patients are driving longer distances and contributing more to CO_2_ emissions while simultaneously experiencing decreased access to high-quality care. Continued expansion and coverage of telemedicine, along with improved broadband access for rural communities under recently passed legislation in US,^25^ will be critical for telemedicine’s continued success and implementation. In addition, at MCC the Virtual Health Department was established to provide clinical and administrative support to patients prior to the telemedicine appointments and developing patient facing and clinician facing educational materials.^7^

Increased CO_2_ emissions are both direct and indirect factors affecting the health of the population. Regarding the direct health implications of climate change, the total disease burden from US health care pollution resulted in a loss of approximately 388 000 disability-adjusted life-years in 2018.^3^ Cancer patients are particularly prone to the direct effects of pollution and particulate matter.^26^ Climate change also results in increased extreme weather events, which affects health care delivery and access to care.^26^ These can result in shifts in care due to diagnostic and treatment delays, lack of access to high-quality care centers, and supply chain disruptions that result in critical shortages of medical supplies and medications.^26^ These factors have been shown time and again to be associated with the survival of cancer patients and increase secondary use of health care resources.^26^ A study modeling the Paris Agreement showed that, if implemented, it would result in significant annual reduction deaths related to pollution, diet, and physical inactivity 2040.^27^

Health Care Without Harm^28^ and members of the Medical Society Consortium on Climate and Health^29^ partnered together to highlight climate change as a health emergency and to call for policymakers to take steps to address it.^30^ For example, the UK’s National Health Service has implemented a mandated government-sponsored carbon reduction initiative for its health system through the Sustainable Development Unit, which tracks GHG progress over time (albeit without including patient travel).^31,32^ A similar US program under the supervision of Department of Health and Human Services may initially help in tracking health care–related GHG emissions and eventually help in reducing them.^3^ A 2021 US Presidential Executive order required federal facilities, notably Veterans Health Administration and Defense Health Agency hospitals and medical facilities, to reduce GHG emissions—certainly a step in the right direction given the large negotiating power the federal government wields.^33^ As our results indicate, telemedicine can help in reducing CO_2_ emissions. Continued support and implementation of telemedicine may assist in meeting these reduction targets.

### Limitations

There are important study limitations that need to be considered. Carbon emissions savings reported in this study are likely to be on the upper end of the estimates. Notably, we assumed all patients traveled via personal automobile to MCC—personal automobiles are known to be higher generators of CO_2_ emissions compared with other modes of public transportation.^13^ Hillsborough County public transit ridership data analysis showed that at the start of the study period (April 1, 2020) public transit ridership was 31% and at the end of the study period (Jun 30, 2021) ridership was 64% of prepandemic levels (eFigure 3 in Supplement 1).^34^ This was consistent with public transit ridership nationally.^35^ Furthermore, given that social isolation protocols were recommended for high-risk patients with active or a history of cancer, comorbidities, and advanced age, it is conceivable that the use of public transportation to attend their oncologic appointments would be even lower. Thus, it stands to reason that the telemedicine visits described in this study were largely shifted from in-person visits that would have been attended using a personal vehicle. While beyond the scope of the study to examine the factors contributing to successful completion of telemedicine vs in-person visits, it is conceivable that not all patients seen by telemedicine would otherwise attend an in-person appointment. In-person cancellation and no-show rates between prepandemic and study period were similar, and so were the in-person and telemedicine cancellation and no-show rates during the study period (eTable 2 in Supplement 1). Thus, the difference in the estimated carbon emission savings is likely to be small in the data set. It is important to note that findings from this study have to be taken in the context of health system and region characteristics and the demographics of their patients. Lastly, we did not account for carbon emissions generated by clinicians commuting to and from work, and we did not factor in electricity used during telemedicine visits.

More generally, while we demonstrate in this study that there are carbon emission savings from telemedicine, further data are needed to examine if long-term oncologic outcomes with telemedicine visits are equivalent to those seen in person. Additionally, the field of telemedicine is in its nascent stages and data are not mature to assess if in-person evaluations can be avoided to truly reduce the carbon emissions. Future studies should assess this specific question of quality provided by telemedicine consultation to better triage the need for in-person evaluation.

## Conclusions

This cross-sectional study highlighted the important benefits of telemedicine and advocate for further implementation of telemedicine in oncology. Telemedicine can help in reducing health care–related carbon emissions.

## Notes

Supplement 1. **eTable 1.** CO_2_ Emission Savings and Equivalencies From Reduced Emissions Due to Telemedicine for Patients Driving ≤60 Minutes and >60 Minutes One-Way **eTable 2.** Number of Cancellations/No Shows for In-person and Telemedicine Visits **eFigure 1.** CO_2_ Emissions Saved From Telemedicine Visits **eFigure 2.** Representation of Number and Percentage of Patients and Their Drive Times to Moffitt Cancer Center From April 1, 2020, to June 30, 2021 **eFigure 3.** Weekly Public Transit Ridership in Hillsborough Area from December 19, 2019, to August 13, 2022

Supplement 2.
Data Sharing Statement
