# Supplementary material for: Estimated Carbon Emissions Savings With Shifts From In-Person Visits to Telemedicine for Patients With Cancer
Source: JAMA Netw Open. 2023 Jan 31;6(1):e2253788. doi: 10.1001/jamanetworkopen.2022.53788 (PMC9890284; doi:10.1001/jamanetworkopen.2022.53788)
Supplement: Supplement 1. — eTable 1. CO2 Emission Savings and Equivalencies From Reduced Emissions Due to Telemedicine for Patients Driving ≤60 Minutes and >60 Minutes One-Way eTable 2. Number of Cancellations/No Shows for In-person and Telemedicine Visits eFigure 1. CO2 Emissions Saved From Telemedicine Visits eFigure 2. Representation of Number and Percentage of Patients and Their Drive Times to Moffitt Cancer Center From April 1, 2020, to June 30, 2021 eFigure 3. Weekly Public Transit Ridership in Hillsborough Area from December 19, 2019, to August 13, 2022 [file jamanetwopen-e2253788-s001.pdf]

Supplemental Online Content

Patel KB, Gonzalez BD, Turner K, et al. Estimated carbon emissions savings with shifts from in-person visits to telemedicine for patients with cancer. *JAMA Netw Open.* 2023;6(1):e2253788. doi:10.1001/jamanetworkopen.2022.53788

eTable 1. CO_2 Emission Savings and Equivalencies From Reduced Emissions Due to Telemedicine for Patients Driving ≤60 Minutes and >60 Minutes One-Way

eTable 2. Number of Cancellations/No Shows for In-person and Telemedicine Visits

eFigure 1. Representation of Number and Percentage of Patients and Their Drive Times to Moffitt Cancer Center From April 1, 2020, to June 30, 2021

eFigure 2. CO_2 Emissions Saved From Telemedicine Visits

eFigure 3. Weekly Public Transit Ridership in Hillsborough Area from December 19, 2019, to August 13, 2022

This supplemental material has been provided by the authors to give readers additional information about their work.

© 2023 Patel KB et al. *JAMA Network Open.*

eTable 1. CO_2 Emission Savings and Equivalencies From Reduced Emissions Due to Telemedicine for Patients Driving ≤60 Minutes and >60 Minutes One Way

CO_2 emission of 411 g/mile was considered midpoint. Estimates corresponding to lower limit (386 g/mile) and upper limit (435 g/mile) are provided as reference.

		**Driving time ≤ 60 minutes**			**Driving time > 60 minutes**		
	**Estimates with g/mile CO_2 emissions**	**386 g/mile**	**411 g/mile**	**435 g/mile**	**386 g/mile**	**411 g/mile**	**435 g/mile**
	**Number of patients**	10 027			13 201		
	**Number of visits**	21 489			27 840		
	**Total miles saved[a]**	1 032 775			6 677 002		
	**Miles saved per visit[a]**<br>Mean (SD)<br>Median (25th – 75th percentile)	48.1 (22.8)<br>49.0 (30.0 – 65.0)			239.8 (133.3)<br>204.0 (148.0 – 302.0)		
	**Total CO_2 kg emissions saved**	398 651	424 471	449 257	2 577 323	2 744 248	2 904 496
	**CO_2 kg emissions saved per visit**<br>Mean (SD)<br>Median (25th – 75th percentile)	18.6 (8.8)<br>18.9 (11.6 – 25.1)	19.8 (9.4)<br>20.1 (12.3 – 26.7)	20.9 (9.9)<br>21.3 (13.1 – 28.3)	92.6 (51.5)<br>78.7 (57.1 – 116.6)	98.6 (54.8)<br>83.8 (60.8 – 124.1)	104.3 (58.0)<br>88.7 (64.4 – 131.4)
**Equivalent greenhouse gas emissions from**	**Passenger vehicles driven for one year**	85.9	91.5	96.8	555	591	626
**Equivalent CO_2 emissions from**	**Gallons of gasoline consumed**	44 858	47 763	50 552	290 010	308 794	7 209 556
	**Homes' electricity use for one year**	77.6	82.6	87.4	501	534	565
	**Homes' energy use for one year**	50.2	53.5	56.6	325	346	366
**Equivalent carbon sequestered by**	**Tree seedlings grown for 10 years**	6 592	7 019	7 429	42 616	45 376	48 026
	**Acres of U.S. forests in one year**	472	502	532	3 050	3 248	3 437

a: round trip

© 2023 Patel KB et al. *JAMA Network Open.*

eTable 2. Number of Cancellations/No Shows for In-person and Telemedicine Visits

April 1, 2018 – Jun 30, 2019 was deemed to be reference period compared to the study period of April 1, 2020 – Jun 30, 2021. Percent In-person/no-show cancellation were determined to be (number of cancellations or no-shows) / (completed visits + cancellation or no-shows).

	April 1, 2018 – Jun 30, 2019	April 1, 2020 – Jun 30, 2021
Number of total visits	304,650	350,782
Number (%) of total cancellations/no-shows	95,582 (23.9%)	119,408 (25.2%)
In-person visits	304,650	301,453
Number (%) of in-person cancellation/no-shows cancellations	95,582 (23.9%)	103,834 (25.6%)
Number (%) of telemedicine visits	Not offered	49,329
Number (%) of telemedicine cancellation/no-shows	N/A	15,574 (24.0%)

© 2023 Patel KB et al. *JAMA Network Open.*

eFigure 1. Representation of Number and Percentage of Patients and Their Drive Times to Moffitt Cancer Center From April 1, 2020, to June 30, 2021

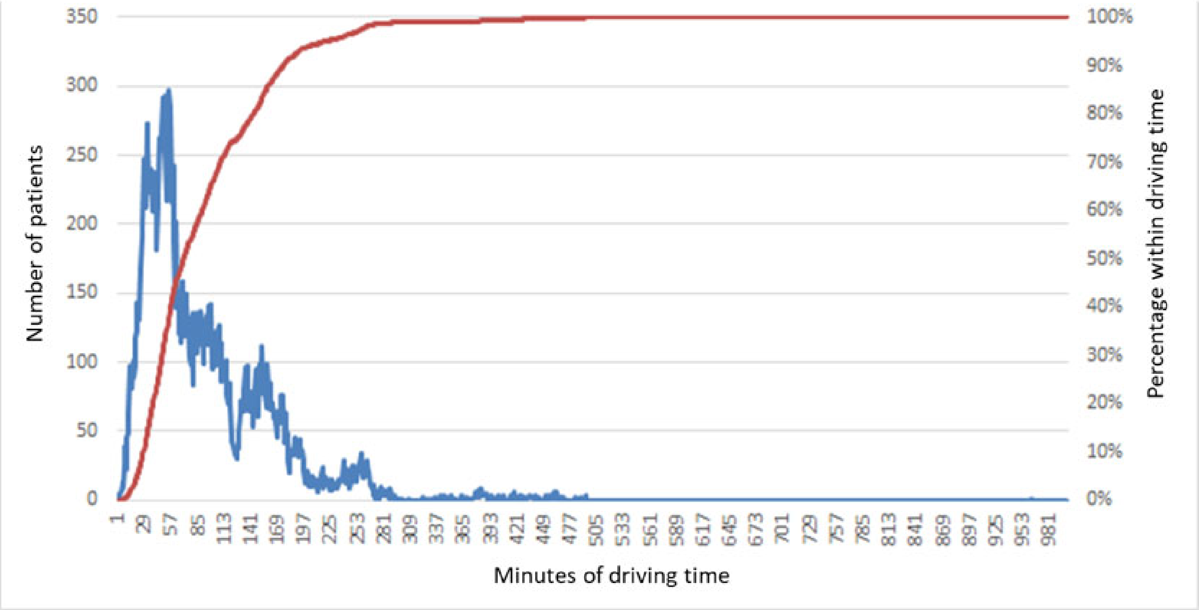

Note: The blue line represents number of patients within certain drive time from Moffitt Cancer Center (MCC). The orange line represents the percentage of patients that are within any given driving time or less. For example, 60% of patients come from 97 minutes or closer. The highest number of patients on a per minute basis occurs within 60 minutes of the facility. This can be noticed both by the high peaks on the blue line and by the steep slope of the orange line.

© 2023 Patel KB et al. *JAMA Network Open.*

eFigure 2. CO_2 Emissions Saved From Telemedicine Visits

Suppl Fig 1: CO_2 emissions saved from telemedicine visits. (A) For patients with driving time ≤ 60 minutes, 10,069 patients had 21,548 visits, which saved 424,471 kg CO_2 emissions (per-visit average savings of 48.1 miles and 19.8 CO_2 kg emissions). (B) For patients with driving time > 60 minutes, 13,232 patients had 27,893 visits, which saved 2,744,248 kg CO_2 emissions (per-visit average savings of 239.8 miles and 98.6 CO_2 kg emissions).

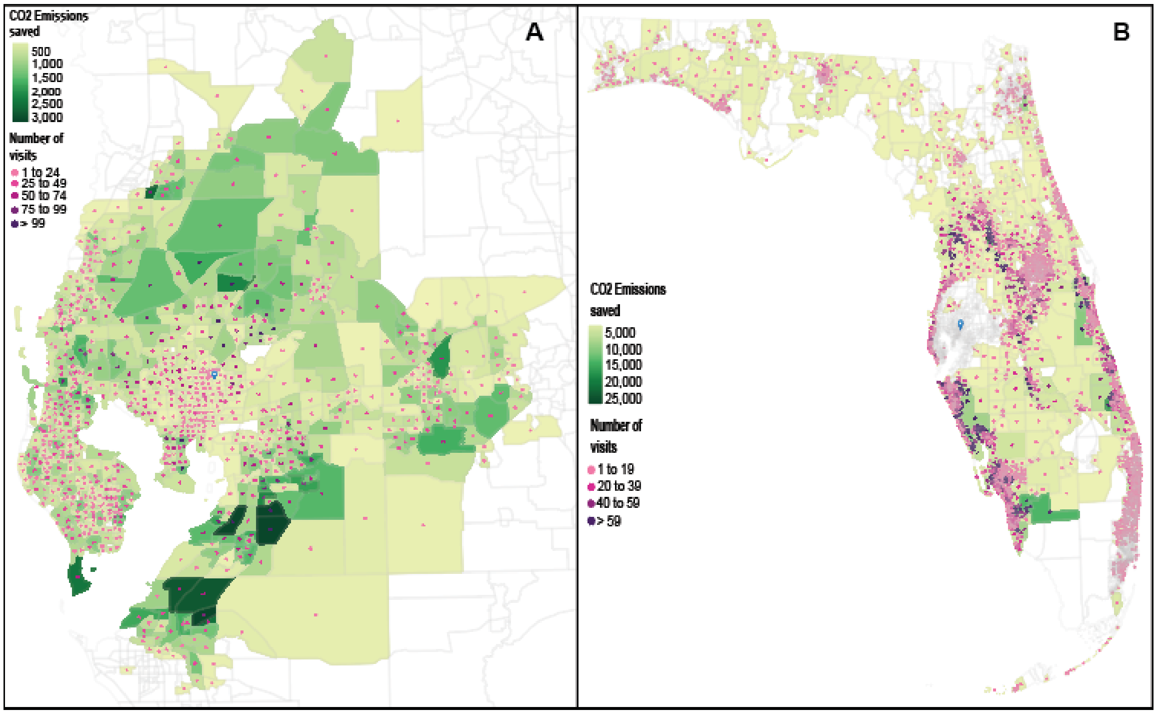

Note: Blule marker represents Moffitt Cancer Center. Geographical boundaries represent census tracts. Census tract centroids were used to map the number of visits per tract. Census tract color represents CO_2 emissions saved, and dot color represents the number of telemedicine visits.

© 2023 Patel KB et al. *JAMA Network Open.*

eFigure 3. Weekly Public Transit Ridership in Hillsborough Area from December 19, 2019, to August 13, 2022

Supplementary Figure 3: Weekly public transit ridership in Hillsborough Area from Dec 19, 2019 to Aug 13, 2022. Public ridership continues to be below pre-pandemic levels. The percentages displayed in the figure represent the percent difference between actual pre-pandemic ridership and estimated ridership for a given period during the pandemic. At the start of the study period (April 1, 2020) public transit ridership was 31%, at the end of the study period (Jun 30, 2021) ridership was 64% and in Aug 7, 2022 it was 85% of pre-pandemic levels.

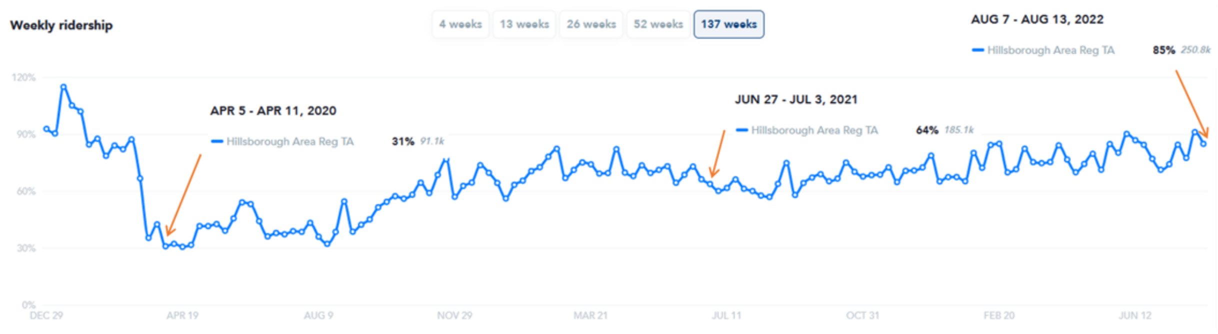

Source: https://transitapp.com/apta

© 2023 Patel KB et al. *JAMA Network Open.*
