# Supplementary material for: Estimated Carbon Emissions Savings With Shifts From In-Person Visits to Telemedicine for Patients With Cancer
Source: JAMA Netw Open. 2023 Jan 31;6(1):e2253788. doi: 10.1001/jamanetworkopen.2022.53788 (PMC9890284; doi:10.1001/jamanetworkopen.2022.53788)
Supplement: Supplement 2. — Data Sharing Statement [file jamanetwopen-e2253788-s002.pdf]

Data Sharing Statement

Patel. Estimated Carbon Emissions Savings With Shifts From In-Person Visits to Telemedicine for Patients With Cancer. *JAMA Netw Open*. Published January 31, 2023.
doi:10.1001/jamanetworkopen.2022.53788

Data
Data available: Yes
Data types: Deidentified participant data
How to access data: krupal.patel@moffitt.org
When available: With publication

Supporting Documents
Document types: None

Additional Information
Who can access the data: Researchers whose proposed use of the data has been approved
Types of analyses: For specified purpose
Mechanisms of data availability: after approval of a proposal and with a signed data access agreement
